# Why Asian Countries are Controlling the Pandemic Better Than the United States and Western Europe

**DOI:** 10.1177/0020731421999930

**Published:** 2021-03-11

**Authors:** Vicente Navarro

**Affiliations:** 1 1466 Johns Hopkins University, Baltimore, MD, USA

**Keywords:** COVID-19, pandemic, neoliberalism, pandemic response

## Abstract

The coronavirus pandemic has shed light on the detrimental impact of neoliberal policies on public health and well-being and as a result, there have been calls for increases in public spending to rectify the lack of public health services. However, neoliberal right-wing parties have dismissed such calls, pointing instead to Asian countries as examples in successfully controlling the pandemic without high public health spending, attributing this to the entrepreneurial orientation of their governments, as opposed to their public services. This article refutes this idea, instead charting the reasons that Asian countries have better controlled the pandemic including prior experience of pandemics, cultural factors, and various successful public health policies. The article concludes by looking at the example of Trump and demonstrating the inadequacies of the business model for dealing with the coronavirus pandemic.

## The Why of the United States and Western Failure

Neoliberal thinking and ideology are declining and on the defensive, as public policies inspired by their dogma have clearly had an enormous, detrimental impact on the quality of life and well-being of the populations who have been exposed to them. (Neoliberalism is a political tradition that is against redistributive policies and favors business interests over labor interests. In the United States, the term “liberal” is used to define progressive policies—the opposite of how it is defined in Europe. This creates considerable confusion. Liberal parties in Europe are right-wing parties and neoliberalism is the extreme form of liberalism. Its major characteristics have been the reduction of public social expenditures and the privatization of health, educational, and social services.) The great dominance that this ideology had in international organizations such as the International Monetary Fund, in forums such as Davos, in the political-media establishments that lead the European Union (and, especially, the Eurozone), and in a large number of governments (mainly those located on both sides of the North Atlantic) is being replaced by other political sensibilities that are trying to reverse regressive labor market reforms (which weakened labor) and their policies of austerity and cuts in public spending (which weakened social protection in the face of the pandemic), giving greater prominence to public interventionism that seeks to put the common good above the interests of the business and financial lobbies that have dominated the public activities of many of these governments in recent years.

A global phenomenon that has accelerated this abandonment of neoliberalism has been the coronavirus pandemic, which has shown in all its crudeness the enormous costs that the application of such policies has had for the majority of those countries, by weakening—through cuts in public spending—public services, such as health care and other services of the Welfare State, which are indispensable for containing such a pandemic. The leadership of the public response over the private one has been, precisely, the result of the enormous limitations of and damage caused by neoliberalism.

## Arguments Given by the Neoliberal Forces

It is not surprising, therefore, that the neoliberal right, supported by large business groups, has mobilized to criticize this new emphasis on the public sphere and the general desire to increase public spending to correct such marked deficits in public services. Their argument is that it is a big mistake to assume there is a need to increase public spending in services, such as health care, for example, because—according to spokespersons of this economic thesis—Asian countries have been more successful than Western countries in controlling the pandemic, despite having much lower public spending on health care and public health and much smaller publicly funded services than, for example, European countries. They therefore claim that the emphasis on spending more on health care and public services is wrong, as public spending is not correlated with a country’s success in solving the problems caused by the pandemic.

They conclude that the key issue is not the size of the public sector, but the quality of management of public services, underlining that Asian countries have states with much lower public spending than in Europe and, in contrast, have lower indicators of infection and mortality of coronavirus because, according to these spokespersons, their states are better led by their political classes and better managed by their public administrations than European countries. They attribute to those Asian states an entrepreneurial culture, common in the world of private business in European countries but perceived to be absent in European public services. The problem lies, therefore, in the low quality of the governmental system, both in the political component and in its administrative and civil service spheres. Therefore, its solution is a deep change of these sectors via the introduction of an entrepreneurial culture, both among politicians (including more entrepreneurs from the private sector in political life) and among civil servants and public administration personnel following the rules of the market.

## How These Neoliberal Arguments are Being Used in Western Europe: The Cases of Italy and Spain

In Spain, the Vox party has been the biggest promoter of neoliberalism. In my presentation to the Working Group on Social Policies in the Commission for Social and Economic Reconstruction of the Spanish parliament, I proposed that higher priority should be given to the expansion of the under-funded Spanish Welfare State, including services such as health and social services and family support services—the fourth pillar of welfare—as well as housing, whose deficits have become apparent during the pandemic. The Vox representative tried to belittle my intervention, stating that what I was asking for was irrelevant to the control of the pandemic in Spain, because Asian countries had done better, achieving lower mortality from the coronavirus than Spain, despite having much lower public spending on health.

In Catalonia, the supporters of such neoliberal thought with the greatest media visibility are economists (some of them known for their proposal to privatize pensions, as General Pinochet did in Chile), such as the senior economic advisor to Davos, widely promoted by the media of the Generalitat de Catalunya (controlled and instrumentalized to the extreme by the successors of pujolism), TV3, and Catalunya Ràdio. These economists, with a very insulting tone, have defined the leftist authorities, and particularly the Spanish government, as inept and incompetent. As proof of this, they point to the authorities’ late response to the pandemic, as well as the great scarcity of masks and protective material, which contrasted with the rapid response of the Asian governments that had abundant protective material from the beginning.

## Let Us Look at the Data, Hidden or Ignored in the Neoliberal Argument

The Asian countries given as an example by the neoliberals are those supposedly representative of the experience of that continent: Singapore, Taiwan, South Korea, and Japan, from which European countries should learn. It is interesting that these countries do not include China or Vietnam, which have indicators as good or even better than those cited. To include them would greatly weaken their argument that the success of these countries is due to the entrepreneurship of their governments, an assertion that the neoliberals are unlikely to make, considering that China and Vietnam are communist regimes in which the public sector plays a clear role, with little or no business management in the health sectors. In reality, the cause of the supposed success of Asian countries—both democratic and dictatorial—has little to do with the entrepreneurial attitude of their states, and much to do with other causes that are not cited and are ignored in the neoliberal arguments.

## Causes, Ignored by the Neoliberals, of the Better Performance of Asian Countries in the Face of Epidemics

One of the most important causes is that these countries have suffered a large number of epidemics in recent years: Asian influenza (H2N2) in 1957, Hong Kong influenza (H3N2) in 1968, avian influenza (H5N1) in 1997, severe acute respiratory syndrome (SARS) in 2003, influenza A (H1N1) in 2009, and the Middle East respiratory syndrome (MERS) coronavirus in 2015. Thus, they have experience and resources available, because the current pandemic is not a new situation for Asia. The last epidemics, such as SARS in 2003 and MERS in 2015, prompted many Asian countries to invest in robust health care and public health infrastructures, which were consequently well equipped to deal with the coronavirus pandemic. They all responded to the pandemic quickly, much more so than Western countries, having had protective resources such as masks in place from the beginning.

In the Western world, countries did not have such experience in responding to this kind of situation. Thus, their response came later and they did not have the materials—such as masks and respirators—that those who had suffered epidemics before very often had. Additionally, countries such as Spain and Italy had seen such resources reduced significantly in the years before the emergence of the pandemic, due to the implementation of neoliberal public policies by their governments. This explains, in part, the high mortality of coronavirus, even among health professionals and workers. In Europe, the country most successful in controlling the pandemic has not been Germany (as was erroneously stated in the TV3 program Preguntes Freqüents by one of the ultra-liberal economists), but Norway, which has a higher public health expenditure than Germany and also than Spain. Of course, there have also been other factors that have influenced each country’s response to the pandemic, but it is incorrect to underestimate the importance of having well-funded and resourced public health services and to refer to Asian countries (which have lower public spending) as proof that this variable is not important.

## Cultural Factors

As I mentioned, other factors are also important. In a large number of the Asian countries mentioned, the use of masks is widespread to protect against respiratory diseases or to protect against the deteriorated environmental conditions in urban centers. This is not the case in Western countries, where masks are not widely used. Add to this poor management by the World Health Organization (WHO), which varied in its proposals after ignoring what the Johns Hopkins University School of Public Health and many other centers of pandemic study had indicated from the beginning. The evidence that the mask protects both the wearer and the person whom they are facing is very well documented. It is surprising that the WHO was slow to recognize airways as a major transmission route, as well as the positive impact of mask use (provided it is appropriate).

## Public Health Measures Were Taken as a Result of Experience With Previous Epidemics, Which are Common in Those Countries

It was also the prior exposure to pandemics that explains why those Asian countries, with the exception of Japan, gave high priority from the beginning of the outbreak to rapid tracing and isolation testing of all cases (and not just the most severe ones), whereas in Europe, these tracing measures were implemented much later. In China, the populations of entire cities were tracked. Additionally, isolations and quarantines were not done so much at home as in institutions, applying innovative surveillance and tracking systems through the use of electronic devices, already widely used in the security services and police (as in South Korea) and not so much in public health. Contact-tracking applications have been installed on a large scale on citizens’ smartphones and are used to locate infected people and those who have been close to them. These and other measures, such as tracking credit card use and images on public surveillance cameras, are also widely used in many of these countries. Such measures appear to have been accepted by the population of the Asian continent, which, I repeat, has experienced several epidemics over a relatively short period of time, creating a culture of solidarity and collective vigilance that other countries—such as the United States and those in Europe—that have not been subjected to such situations in their recent past might be reluctant to accept.

In addition to these differentiating factors of Asian countries, other elements have contributed to the success of some of these countries, such as their insularity and easy control of their borders, as has been the case with Taiwan. But for all of them, belonging to a continent with extensive experience in epidemics has socialized the population and society to respond quickly and effectively to the danger of this coronavirus pandemic.

The Asian experience does not, therefore, demonstrate that well-resourced and funded health care services (including public health services) are a low value or even irrelevant variable, nor is it likely that the best control of the pandemic is the result of public services being managed and directed politically under a business culture favorable to following the sacrosanct laws of the market. In fact, there exists a great diversity of management systems of public services in the Asian continent, including those that have almost opposite cultures yet are equally or more successful than those defined as entrepreneurial by the neoliberals. The crux of the matter is that all these countries have had many more epidemics than Western countries, which has given them more experience in how to handle them. And this is without discounting cultural variables and common experience, which have little to do with the arguments promoted by the neoliberal authors. To evaluate the impact of a public policy in one country, one cannot use the impact of the same policy in another country without first comparing the economic, political, cultural, and social context of both, which is something not done by the neoliberals, who incorrectly assume that this context in the countries on both sides of the North Atlantic is comparable with that of the Asian countries.

## Failure to Act From a Business Perspective on the Pandemic: The Trump Case

Another widely held position among the proponents of neoliberalism is that economic activity should be prioritized above all else, avoiding by all means home quarantine. The best example of this position has been President Trump, the maximum expression of nationalist ultra-liberalism. What this argument seems to forget is that without control of the pandemic, there will be no economic recovery. In reality, the Trump administration has shown where the introduction of the entrepreneurial spirit into public administration leads: to enormous inefficiency (as well as corruption) and a questioning and confrontation with scientific societies and experts in public health. This has led to a situation in which a country like the United States, which represents only 4% of the entire world population, represents almost 19% of all deaths from coronavirus. Furthermore, the United States has shown the enormous inefficiency of the business model in the management of health care, most of which is privatized. It is the country that spends the most on health (about 17% of its gross domestic product, mostly private spending) and, in contrast, is the country that has one of the poorest health indicators in the developed world (according to the United Nations Human Development Report, the United States is among the countries with the worst health indicators among developed countries, number 38 out of a total of 40), with the greatest number of people without health care and with a greater percentage of the population wanting to change the business management system of the health system. It is a system with excellent professionals and health centers, but the financing and management controlled by private insurance companies is a disaster. Even Republican President Nixon defined it as such. The pandemic has clearly shown the enormous inadequacies of such a business model.

